# Hemoperitoneum complicating uterine fibroid: case report

**DOI:** 10.1093/jscr/rjaf095

**Published:** 2025-03-05

**Authors:** Charles Mawunyo Senaya, Francis Jojo Moses Kodzo Damalie, Kwaku Gyimah Amoa-Gyarteng, Humphrey Oduro Tweneboah, Derrick Osei Asamoah, Augustine Tawiah

**Affiliations:** Directorate of Obstetrics and Gynecology, Komfo Anokye Teaching Hospital, PO Box KS 1934, Kumasi, Ghana; Department of Obstetrics and Gynecology, School of Medical Sciences, College of Health Sciences, Kwame Nkrumah University of Science Technology, Kumasi, Ghana; Directorate of Obstetrics and Gynecology, Komfo Anokye Teaching Hospital, PO Box KS 1934, Kumasi, Ghana; Department of Obstetrics and Gynecology, School of Medical Sciences, College of Health Sciences, Kwame Nkrumah University of Science Technology, Kumasi, Ghana; Department of Obstetrics and Gynecology, School of Medical Sciences, College of Health Sciences, Kwame Nkrumah University of Science Technology, Kumasi, Ghana; Department of Obstetrics and Gynecology, School of Medical Sciences, College of Health Sciences, Kwame Nkrumah University of Science Technology, Kumasi, Ghana; Department of Obstetrics and Gynecology, School of Medical Sciences, College of Health Sciences, Kwame Nkrumah University of Science Technology, Kumasi, Ghana; Directorate of Obstetrics and Gynecology, Komfo Anokye Teaching Hospital, PO Box KS 1934, Kumasi, Ghana; Department of Obstetrics and Gynecology, School of Medical Sciences, College of Health Sciences, Kwame Nkrumah University of Science Technology, Kumasi, Ghana

**Keywords:** hemoperitoneum, uterine fibroid complications, Kumasi, Ghana, sub-Saharan Africa

## Abstract

Hemoperitoneum due to bleeding from a vessel on a uterine fibroid is very rare. We report a case of hemoperitoneum in a 32-year-old Ghanaian woman who collapsed due to hemoperitoneum from a bleeding vessel on a pedunculated fibroid. Urgent resuscitation followed by laparotomy for myomectomy was performed successfully. Clinicians especially in sub-Saharan Africa where uterine fibroids are endemic should be aware of this rare complication of fibroids to avoid delay in diagnosis and to prevent morbidity and mortality.

## Introduction

Uterine fibroids are the commonest gynecological tumor in sub-Saharan Africa; however, acute complication requiring urgent surgical intervention is rare [1, 2]. An acute and life-threatening complication of uterine fibroids is the spontaneous rupture of blood vessels overlying the fibroid [3]. This may lead to hypovolemic shock and may be rapidly fatal. Early diagnosis and urgent surgical intervention are required to avoid mortality.

## Case report

A 32-year-old nullipara presented with sudden onset of abdominal pains, dizziness, and collapse with temporary loss of consciousness. She regained consciousness while being transported to the hospital. She had previously been diagnosed to have uterine fibroid that was asymptomatic. She had no other significant past medical history. She was a nurse and not married. Clinical examination revealed a fully conscious lady in severe pain. She was pale, anicteric, and afebrile. Her BP was 84/56 mmHg with a pulse rate of 124/min.

**Figure 1:**
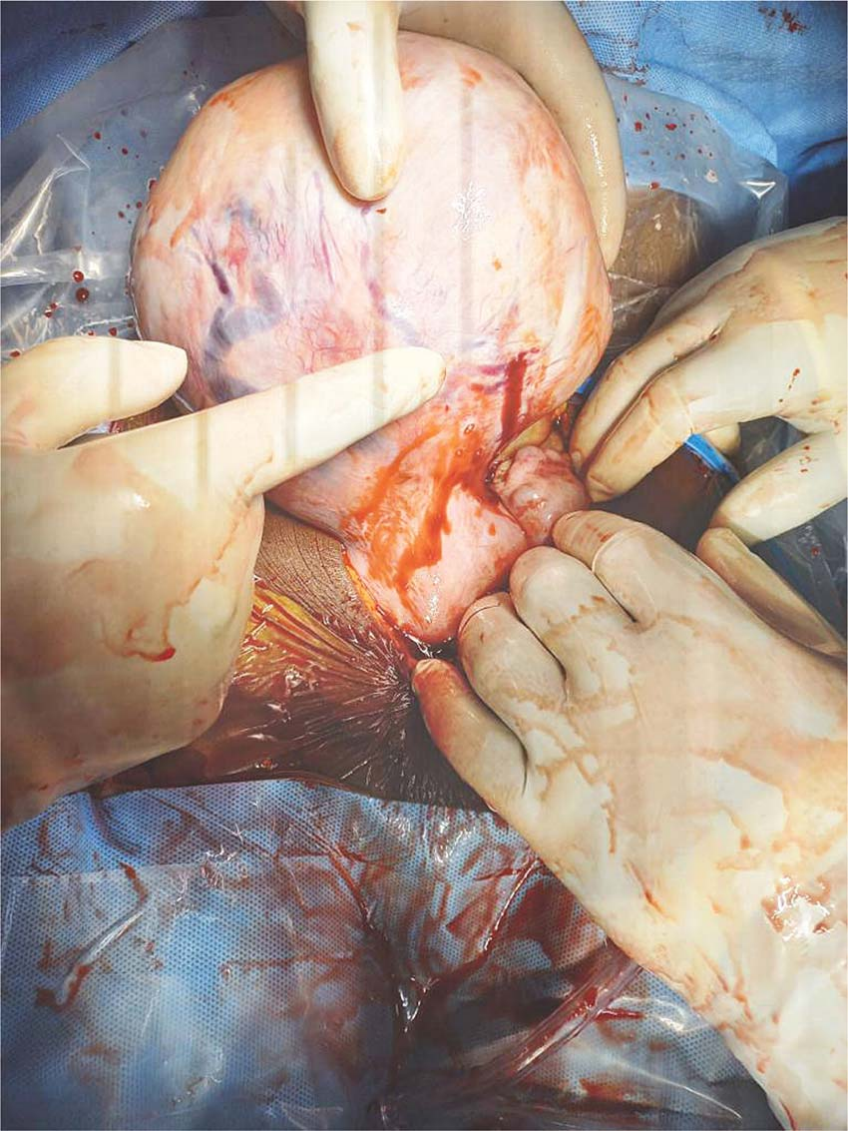
Predunculated fibroid with overlying bleeding vessels.

Her abdomen was distended and tender. There was a multinodular abdomino-pelvic mass equivalent to about 26 weeks’ pregnant uterus. An abdominopelvic ultrasound showed a massive intraperitoneal fluid and an enlarged uterus with multiple myomas, the largest noted at the fundus measuring 17.8 × 13.0 × 15.5 cm^3^. Her hemoglobin level was 8.0 g/dl and her platelet count was 279 × 10^3^/ul. The urinary pregnancy test was negative. We initiated resuscitation with colloids and transfused two units of whole blood. An emergency laparotomy was performed. During the laparotomy, we identified a huge pedunculated myoma with an active bleeding vessel overlying it (Fig. 1) as the source of the hemoperitoneum, which totaled about 4 L. Both ovaries and fallopian tubes were normal. Her postoperative recovery was unremarkable. She was discharged on postoperative Day 5.

## Discussion

Hemodynamic instability due to hemoperitoneum resulting from bleeding vessels overlying a uterine fibroid may present a diagnostic dilemma, especially when the pregnancy test is negative in a reproductive-age woman. It is important for gynecologists to consider this rare complication of uterine fibroids, particularly in sub-Saharan Africa where uterine fibroids are endemic so that such cases are diagnosed early and promptly managed to avoid fatality. The mechanism of a blood vessel overlying a uterine fibroid to rupture had not been clearly elucidated. Bleeding may be venous or arterial. While venous bleeding has been attributed to torsion of a pedunculated fibroid or pregnancy causing congestion resulting in venous rupture [4], arterial bleeding may be associated with systemic hypertension [5]. Abdominal trauma resulting in rupture and bleeding of uterine fibroid have also been reported [2, 6]. Our patient did not have any of these risk factors. An intraabdominal mass associated with abdominal pains, hemoperitoneum, and hypovolemic shock is often the clinical presentation [3, 7] as was in our case. Abdominopelvic ultrasound scan is valuable in the diagnosis of hemoperitoneum though cannot identify the source of bleeding. Contrast-enhanced CT scans could be useful in determining the source of bleeding [7]; however, arranging and transporting patients for CT scan, especially in developing countries, may delay surgical intervention. Clinical examination and ultrasound identification of hemoperitoneum are sufficient for preoperative diagnosis [1]. Timely resuscitation including hemotransfusion and urgent surgical intervention for either hysterectomy or myomectomy is the only definitive treatment [8]. Myomectomy is indicated in women who desire to retain fertility [9]. Myomectomy was performed in our case with a successful outcome.

## Conclusion

Hemoperitoneum complicating uterine fibroids is very rare, however, given the high prevalence of uterine fibroids, especially in sub-Saharan Africa, this life-threatening condition must be kept in mind in women with fibroids with hemoperitoneum to avoid mortality.

## References

[1] Daimon A, Tanaka T, Kogata Y, et al. Hemoperitoneum associated with uterine fibroids: a case report. Medicine 2021;100:e24024–4. 10.1097/MD.0000000000024024. 33725815 PMC7969270

[2] Elkbuli A, Shaikh S, McKenney M, et al. Life-threatening hemoperitoneum secondary to rupture of a uterine leiomyoma: a case report and review of the literature. Int J Surg Case Rep 2019;61:51–5. 10.1016/j.ijscr.2019.07.004. 31326857 PMC6642253

[3] Lim WH, Cohen SC, Lamaro VP. Intra-abdominal haemorrhage from uterine fibroids: a systematic review of the literature. BMC Surg 2020;20:70. 10.1186/s12893-020-00736-5. 32293414 PMC7157977

[4] Dahan MH, Ahmadi R. Spontaneous subserosal venous rupture overlying a uterine leiomyoma. A case report. J Reprod Med 2002;47:419–20. 12063882

[5] Schwartz M, Powell K. Spontaneous rupture of a leiomyoma causing life-threatening intra-abdominal hemorrhage. Case Rep Obstet Gynecol 2017;2017:3701450. 10.1155/2017/3701450. 28127487 PMC5239864

[6] Oride A, Kanasaki H, Hara T, et al. Hemoperitoneum from ruptured vein overlying a uterine myoma: a case report and review of the literature. CEOG 2019;46:337–9. 10.12891/ceog4503.2019.

[7] Maduanusi C, Balachandran S, Sathiyathasan S, et al. Painless spontaneous haemoperitoneum secondary to a uterine leiomyoma/fibroid: unusual presentation of a life-threatening differential. BMJ Case Rep 2021;14:e243465. 10.1136/bcr-2021-243465. PMC8491287 34607815

[8] Lotterman S . Massive hemoperitoneum resulting from spontaneous rupture of uterine leiomyoma. Am J Emerg Med 2008;26:974.e1–2. 10.1016/j.ajem.2008.02.029. 18926384

[9] Gulati N, Raman S, Srinivasan M, et al. Rare gynaecological emergency: massive intraperitoneal haemorrhage from spontaneous rupture of a superficial vessel on a large leiomyoma. BMJ Case Rep 2016;2016:bcr2015212576. 10.1136/bcr-2015-212576. PMC4735157 26825935

